# In Vitro Evaluation of the Antibacterial Effect and Influence on the Bacterial Biofilm Formation of Glutamic Acid and Some Structural Analogues

**DOI:** 10.3390/antibiotics14040415

**Published:** 2025-04-19

**Authors:** Octavia-Laura Oancea, Cristina Nicoleta Ciurea, Anca Delia Mare, Adrian Man, Ruxandra Stefanescu, Aura Rusu

**Affiliations:** 1 Organic Chemistry Department, Faculty of Pharmacy, George Emil Palade University of Medicine, Pharmacy, Science, and Technology of Targu Mures, 540142 Targu Mures, Romania; octavia.moldovan@umfst.ro; 2 Microbiology Department, George Emil Palade University of Medicine, Pharmacy, Science, and Technology of Targu Mures, 540142 Targu Mures, Romania; anca.mare@umfst.ro (A.D.M.); adrian.man@umfst.ro (A.M.); 3 Pharmacognosy and Phytotherapy Department, Faculty of Pharmacy, George Emil Palade University of Medicine, Pharmacy, Science, and Technology of Targu Mures, 540142 Targu Mures, Romania; ruxandra.stefanescu@umfst.ro; 4 Pharmaceutical and Therapeutic Chemistry Department, Faculty of Pharmacy, George Emil Palade University of Medicine, Pharmacy, Science, and Technology of Targu Mures, 540142 Targu Mures, Romania; aura.rusu@umfst.ro

**Keywords:** glutamic acid, monosodium glutamate, glutamine, glutamic acid diethyl ester, amino acids, antibacterial effect, biofilm formation

## Abstract

**Background/Objectives**: Glutamic acid (GLA) is an essential amino acid with a key role in human metabolism. A potential involvement in anticancer therapy and possible antibacterial and anti-biofilm effects were also observed. Glutamine (GLN) and monosodium glutamate (MSG) are GLA structural derivatives for which the last two effects were evaluated, with contradictory results. Therefore, this study aimed to assess the antibacterial activity and the influence on the biofilm formation of GLA, GLN, MSG, and glutamic acid diethyl ester (GLADE) on clinically relevant bacteria. **Methods**: Gram-positive and Gram-negative bacterial reference strains were used to test the antibacterial and anti-biofilm effects of GLA, GLN, MSG, and GLADE. The antibacterial properties were assessed by detecting the minimum inhibitory concentration (MIC) and the minimum bactericidal concentration (MBC). The influence on biofilm formation was assessed by the crystal violet method, reading the optical densities (ODs) by spectrophotometry. **Results**: GLN did not demonstrate an inhibitory capacity at the maximum tested concentration (2.86 mg/mL); GLA showed inhibitory activity at 1.76 mg/mL and 0.88 mg/mL; MSG inhibited the growth of all bacterial strains at 112 mg/mL; GLADE had the most promising results on all bacterial strains (MICs of 12.75 mg/mL and 25.5 mg/mL). GLADE showed satisfactory MBC values on all bacterial strains (at 51 mg/mL and 25.5 mg/mL). **Conclusions**: GLA and some structural analogues are attractive options for possible antibacterial activity; optimizing GLADE to increase its antibacterial activity could be a new approach.

## 1. Introduction

Glutamic acid (GLA) and its derived amide, glutamine (GLN), are amino acids with multiple metabolic implications in the human organism. The biochemical relationship and the complexity of their metabolic pathways are the basis of the attempts to expand their biological effect. The well-known metabolic interrelationships of GLA, GLN, and monosodium glutamate (MSG) addressed in the scientific literature and our previous studies [1,2] contributed to a focus on new possible effects (e.g., the possible anticancer effect of some GLA structural analogues [3,4] or the potential toxicity of MSG [1]).

Considering the documented potential of amino acids, more precisely GLA, to expand their well-known role, our interest in deepening this topic has grown. Among their other possible effects, the potential antibacterial activity and the influence on biofilm formation were also targeted [5,6]. This topic is controversial–on one side, positive results were obtained for the antimicrobial effect of some amino acids, and their synergistic effect with other antibiotics is promising [6,7,8,9]; on the other hand, these amino acids are essential for the functional integrity of bacteria. Amino acids are valuable nutritional sources for the bacterial cell, contributing to its survival and biofilm development [10]. Thus, new contributions to this topic are welcome to clarify and complete some uncertain aspects.

**Figure 1:**
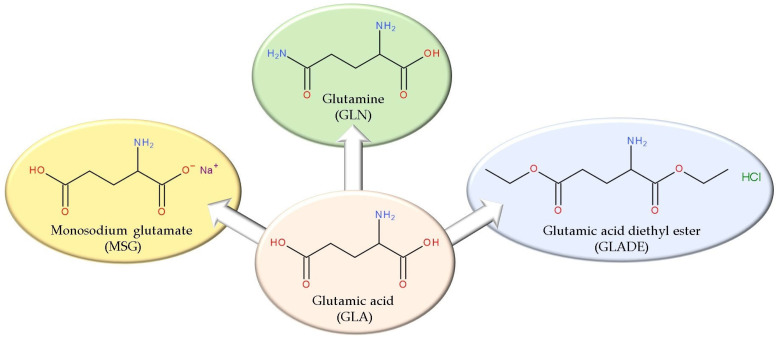
The chemical structures of glutamic acid and its structural analogues (monosodium glutamate, glutamine, and glutamic acid diethyl ester hydrochloride) targeted in this study.

Also, our previous publications [3,4] highlighted the promising anticancer potential of some GLA derivatives and created a shift in direction toward other possible effects of these derivatives, which have been studied less in the scientific literature. One such compound is glutamic acid diethyl ester (GLADE) (Figure 1), for which, to our knowledge, no information related to the potential antimicrobial effect is published in the scientific literature.

Therefore, the objective of this study is the in vitro evaluation of the potential antibacterial effect of GLA and some structural analogues (GLN, MSG, and GLADE) by determining the minimum inhibitory concentration (microdilution method) and the minimum bactericidal concentration, as well as their influence on the bacterial biofilm formation of several Gram-positive and Gram-negative bacteria.

Since data on the influence of GLA and GLN on biofilm development are contradictory, we aim to contribute new results to confirm or refute their ability to inhibit biofilm formation. The results can lead to a better understanding and clarification of the importance and role of amino acids in bacterial survival. Also, we intend to bring new relevant data on the possible antibacterial and anti-biofilm effects of MSG and GLADE. These two analogues were poorly approached in experimental studies.

Therefore, our study could open up new research directions in the field by addressing several key aspects: the evaluation of two compounds (GLA and GLN) that are commonly included in such studies, particularly those focused on their synergistic effects with other antibiotics, rather than their individual effects; the analysis of the impact of a controversial compound (MSG) and a compound with limited coverage in the scientific literature (GLADE); the assessment of the antibacterial effect and the influence on biofilm formation of the four compounds on clinically relevant Gram-positive and Gram-negative bacteria.

## 2. Results

### 2.1. Evaluation of the Antibacterial Effect (Minimum Inhibitory Concentration (MIC) and Minimum Bactericidal Concentration (MBC) Determination)

The results of the minimum inhibitory concentration (MIC) determination by the microdilution method and the minimum bactericidal concentration (MBC) determination are presented in Table 1 and Table 2. After performing the MIC determinations, bacterial growth was observed in all GLN-containing wells; for this reason, this compound was not included in the MBC evaluation. Similar results are observed for GLA for *Klebsiella pneumoniae* and *Escherichia coli*. The MIC values for GLA are either at the highest concentration tested or the second highest, while the MBC values exceed the maximum concentration. MSG’s MIC value corresponds to the maximum concentration selected, and the MBC exceeds it for all six bacterial strains. For GLADE, the MIC value is generally the fourth highest concentration (12.75 mg/mL, corresponding to 12.5% of the maximum concentration) for most bacterial strains, except for *K. pneumoniae* and *E. coli*, where the MIC is 25.5 mg/mL, corresponding to 25% of the maximum concentration. Regarding the MBC values for GLADE, the results show that 25% of the maximum concentration (25.5 mg/mL) is typically required for bactericidal activity, except for MSSA, where 50% of the maximum concentration (51 mg/mL) is needed.

### 2.2. Evaluation of the Influence of the Four Compounds on Biofilm Formation

**Figure 2:**
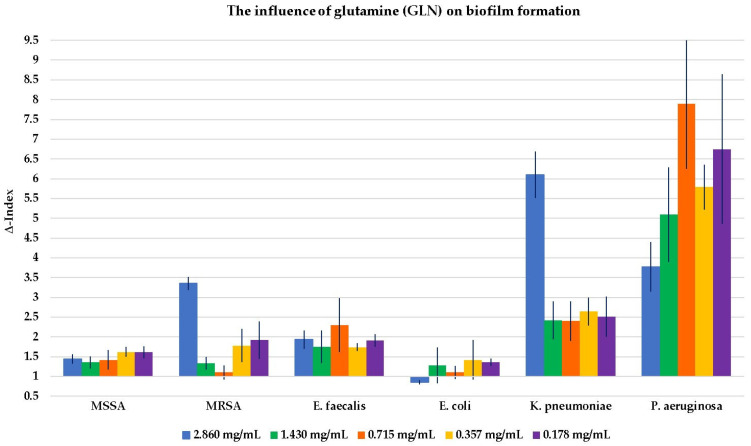
The influence of glutamine (GLN) on biofilm formation in all six Gram-positive and Gram-negative bacterial strains, based on GLN concentration. The results are presented as Δ-Index ± standard deviation (Δ-Index being the ratio between the sample mean extinction and the positive control mean extinction).

**Figure 3:**
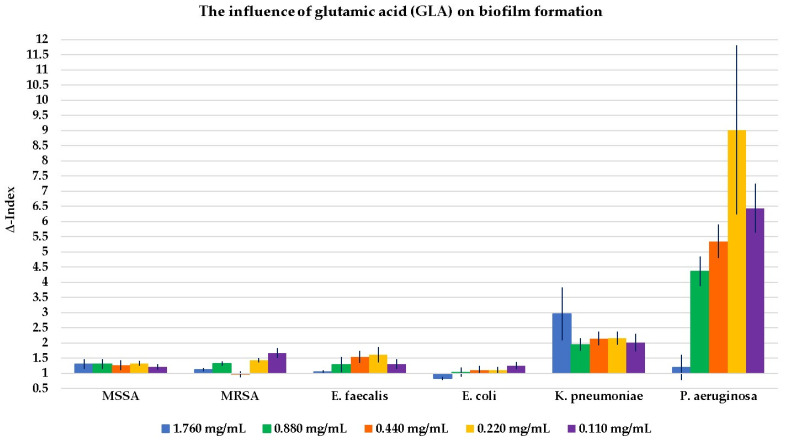
The influence of glutamic acid (GLA) on biofilm formation in all six Gram-positive and Gram-negative bacterial strains, based on GLA concentration. The results are presented as Δ-Index ± standard deviation (Δ-Index being the ratio between the sample mean extinction and the positive control mean extinction).

**Figure 4:**
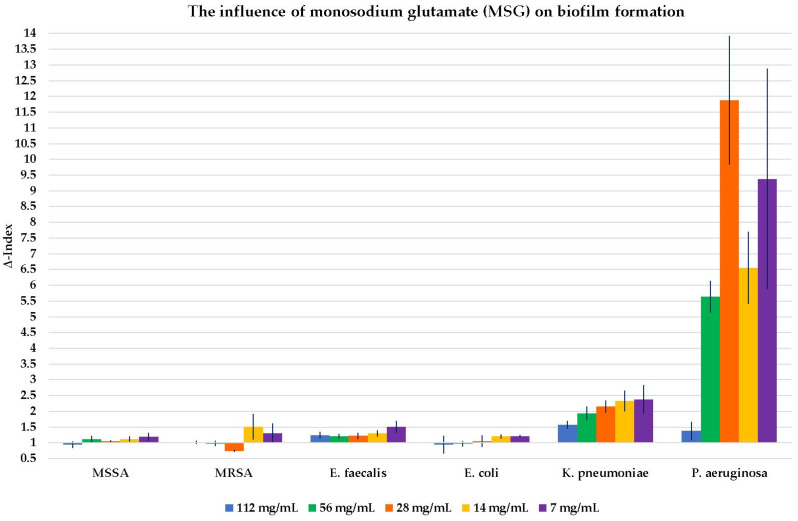
The influence of monosodium glutamate (MSG) on biofilm formation in all six Gram-positive and Gram-negative bacterial strains, based on MSG concentration. The results are presented as Δ-Index ± standard deviation (Δ-Index being the ratio between the sample mean extinction and the positive control mean extinction).

**Figure 5:**
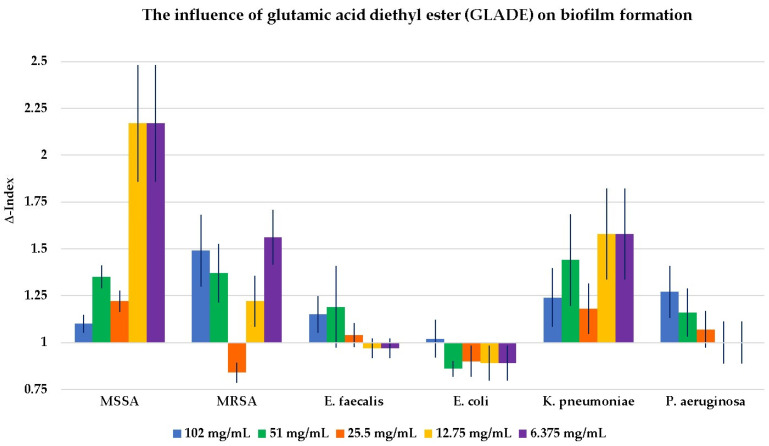
The influence of glutamic acid diethyl ester (GLADE) on biofilm formation in all six Gram-positive and Gram-negative bacterial strains, based on GLADE concentration. The results are presented as Δ-Index ± standard deviation (Δ-Index being the ratio between the sample mean extinction and the positive control mean extinction).

The results of the influence of GLN, GLA, MSG, and GLADE on the biofilm formation of all six selected bacterial strains are shown in Figure 2, Figure 3, Figure 4 and Figure 5. Additionally, tables presenting all the results corresponding to the graphical representations in Figure 2, Figure 3, Figure 4 and Figure 5, shown as mean ± standard deviation, are included in the Supplementary Material (Tables S1–S4).

GLN, GLA, and GLADE generally enhanced the biofilm formation ability of MSSA, while MSG did not demonstrate any effect on this bacterial strain’s biofilm. The same effect was observed for GLN, GLA, and GLADE on MRSA; MSG did not influence MRSA biofilm formation at higher concentrations. However, it had a slightly inhibiting activity at 25% concentration (28 mg/mL) and a stimulating effect at lower ones. GLN and GLA generally stimulated the biofilm formation of *E. faecalis*, while MSG presented this effect only at lower concentrations, and neither negative nor positive effects were observed for GLADE. A general tendency not to influence the biofilm formation of *E. coli* (by stimulation or inhibition) by any of the four compounds was observed, with a few exceptions (especially for GLN). In contrast with these results, a generally stimulating activity by the four compounds on *K. pneumoniae* biofilm development was observed. The same characteristic was seen for *P. aeruginosa*, with a few exceptions, especially for GLADE (a lack of influence on the bacterial biofilm for the 50–6.25% concentration range (51 mg/mL; 25.5 mg/mL; 12.75 mg/mL; 6.375 mg/mL)).

### 2.3. In Silico Evaluation of Glutamic Acid Diethyl Ester’s (GLADE’s) Properties as a Potential Drug Candidate

The positive results of GLADE’s antibacterial activity led to the in silico analysis and theoretical prediction of several specific properties, defining its profile and contributing to new findings that could further highlight the potential of this compound. Based on GLADE’s chemical structure, Table 3 presents a characterization of some physicochemical properties and predictions of pharmacokinetic properties, drug-likeness and lead-likeness characteristics, possible antibacterial activity, and toxicity assessed theoretically using computational methods.

## 3. Discussion

### 3.1. The Antibacterial Effect (Minimum Inhibitory Concentration (MIC) and Minimum Bactericidal Concentration (MBC) Determination)

The MIC determination performed in our study for GLN indicated that a higher concentration than the maximum tested one is needed to exhibit a possible inhibitory effect on bacterial growth for all tested strains. GLN did not show remarkable antibacterial effects in the dose range chosen in this study. However, in similar doses, GLN has been reported to increase the effectiveness of some co-administered antibiotics (gentamicin and *L*-GLN in the case of MRSA, *Listeria monocytogenes*, and *Corynebacterium diphtheriae*–Gram-positive bacteria) [8]. The GLN–ciprofloxacin combination has also been reported to have a synergistic effect in a study involving mice with septic shock induced by *P. aeruginosa*. The administration of the GLN–ciprofloxacin combination led to the following effects compared to an untreated control group: the increased survival rate of experimental animals, an increase in the serum levels of interleukin IL10 (inhibitor of some pro-inflammatory cytokine production) and of the heat shock protein HL-70 (with a role in decreasing the inflammatory response and cell preservation in high stress), decreased TNF-alpha serum levels, and liver necrosis severity [11]. Additionally, when *L*-GLN was combined with gentamicin, it was suggested that *L*-GLN might have disrupted the ΔpH and increased cell membrane permeability, which enhanced the uptake of gentamicin, leading to bacterial death. At the same time, *L*-GLN treatment reduced reactive oxygen species levels through glutathione, thereby increasing the sensitivity of MRSA to gentamicin [8].

Encouraging MIC determination results were observed for GLA in the present study, especially for Gram-positive bacteria, highlighting the concentration of 0.88 mg/mL for MRSA. For MSSA and *E. faecalis*, a concentration of 1.76 mg/mL was needed to demonstrate the inhibitor effect of GLA. The same concentration (1.76 mg/mL) was sufficient for the growth inhibition of *P. aeruginosa* but not for other Gram-negative bacteria.

While some experimental studies in the scientific literature demonstrate the antibacterial effects of GLA or GLN, either alone or in combination with traditional antibiotics, the independent mechanism by which they exert their antibacterial action is poorly understood. The antibacterial effects of several amino acids were documented for different Gram-positive and Gram-negative bacterial strains. An example in which the structure of *D*-GLA was exploited for this purpose is the synthesis and evaluation of an inhibitor (based on the structure of this amino acid) of MurD and MurE ligases involved in the peptidoglycan synthesis of *E. coli* and *S. aureus*. The inhibition of MurD in *E. coli* and *S. aureus* was observed at half-maximal inhibitory concentration (IC50) values of 8.2 and 6.4 μM and of MurE at IC50 values of 180 and 17 µM [12]. Antimicrobial agents based on the structure of some amino acids are also reported by Nowak et al. (2021) [13].

A mixture of *D*-Cysteine, *D*-Aspartic acid (*D*-Asp), and *D*-GLA and a mixture of *L*-Cysteine, *L*-Aspartic acid (*L*-Asp), and *L*-GLA at a concentration of 40 mM can prevent the growth of *Streptococcus mutans*. *D*-amino acids are an essential component of the cell wall peptidoglycan. During its synthesis, the incorporation of exogenous *D*-amino acids into the peptide side chains, replacing the terminal *D*-alanine, can directly impact the cross-linking of glycan strands, thereby influencing the strength and flexibility of the peptidoglycan. Additionally, *D*-amino acids are not typical metabolites in bacterial cells, so introducing exogenous *D*-amino acids may disrupt normal cellular metabolism [6,7]. In another study, it was concluded that when a mixture of amino acids, including GLA, was tested for its effects on the antibacterial functions of human peripheral blood neutrophilic granulocytes, the amino acids enhanced the bactericidal activity of neutrophils against *S. aureus* at a neutrophil–bacteria ratio of 10:1 [14].

At the same time, exploiting the synergistic effect of *L*-GLA or a mixture of amino acids in combination with other antibacterial compounds can lead to favourable results [6]. Also, a mix of *D*-amino acids, including *D*-GLA, along with antibiotics such as gentamicin or amoxicillin, led to a decrease in the dose of the antibiotic (increasing the potency) to achieve the antibacterial effect [7]. Depending on the optical activity, there could be differences in the antibacterial effects of amino acids, but additional studies are needed to clarify this aspect. Similar effects were observed in studies addressing this topic for *D*- and *L*-amino acids [15].

Polygamma-glutamic acid (PG) was also included in studies to evaluate its antibacterial effect. A dose of 150 mg/mL of PG was tested (through a disc diffusion test) on a series of pathogens, with subsequent MIC determination. The most pronounced effect was observed against *S. aureus* and *L. monocytogenes*, while the Gram-negative bacteria presented resistance. The best results were obtained against *S. aureus* with a MIC value of 16.125 mg/mL [16]. PG with different molecular masses was also tested against the *E. coli* bacteria; antibacterial activity was demonstrated with the MIC values decreasing with an increase in the molecular mass of PG (while maintaining the same culture conditions). It was concluded that the antibacterial effect is exerted by damaging the microbial cellular structure, primarily the cell wall [17]. PG’s hydrophilic and anionic nature may explain its antibacterial activity since hydrophobic interactions between the cell wall and a substrate would enhance microbial adhesion, subsequently leading to bacterial proliferation [18].

Antimicrobial peptides are a class of small peptides that widely exist in nature, usually consisting of 12–100 amino acid residues; they display rapid and potent antimicrobial activity against various pathogens, such as bacteria, fungi, parasites, and viruses [19,20]. They play a crucial role as effector molecules in the innate immune systems of both prokaryotic and eukaryotic organisms. Antimicrobial peptides could exert their microbicidal effects by disrupting the microbial cell membrane but also through intracellular action. Anionic peptides, often rich in GLA and aspartic acid, generally contain five to seventy amino acid residues and carry a net charge of −1 or −2. Studies have shown that these anionic peptides exhibit enhanced potency against Gram-positive and Gram-negative bacteria when combined with synergistic cationic antimicrobial peptides and Zn^2+^. The cationic peptides facilitate a cationic linkage between the anionic peptide and the microbial cell membrane, allowing the anionic peptide to be transported to intracellular targets without causing damage to the membrane structure. These anionic peptides primarily target ribosomes within the cell, inhibiting ribonuclease activity, which leads to microbial cell death [19].

MSG showed similar effects on the six bacterial strains at the maximum concentration tested for the MIC determination (112 mg/mL). Although the range of concentrations tested in this study for MSG is increased compared to those of GLN and GLA, this range was chosen based on experimental study data in which the concentration of MSG subjected to successive dilutions was 100 mg/mL (a concentration close to the one used in our study). The selected Gram-negative bacteria in our study coincide with those in the study of Bhuvaneswari et al. (2015) [5]. However, in their experiment, the inhibitory activity of MSG was observed only on *P. aeruginosa* (at the concentration of 100 mg/mL). Among the Gram-positive bacteria considered in our study, *S. aureus* was also evaluated by Bhuvaneswari et al. (2015) [5], but no positive results were found in their case.

Among the tested compounds, GLADE provided promising results for the MIC value for all tested bacterial strains. GLADE inhibited all Gram-positive bacterial strains’ activity at 12.75 mg/mL. The same results were obtained for *P. aeruginosa*; a 25.5 mg/mL concentration of GLADE had an inhibitory effect on other Gram-negative bacterial strains. Although GLADE is a structurally simple derivative of GLA, there is limited detailed information in the scientific literature regarding its potential biological effects, particularly its antibacterial properties. Our findings suggest that GLADE could act as a non-NMDA antagonist of GLA [21]. GLADE is considered a partially effective antagonist of glutamate-induced neuronal excitations. It is particularly effective in the hippocampus, inhibiting GLA’s action and reducing evoked field potentials. Given the hippocampus’ crucial role in certain forms of learning, these findings led to a hypothesis that GLADE may interfere with learning processes [22]. Therefore, although no recent data are available on the antibacterial effect of GLADE, the potential antagonistic relationship between GLADE and GLA may provide a foundation for future explanations of this activity; however, further studies are necessary.

GLN was excluded from the MBC evaluation because it did not inhibit bacterial growth even at the highest tested concentration; this phenomenon also occurred in the case of GLA for *K. pneumoniae* and *E. coli* strains. Although GLA showed positive results of bacterial inhibition for the concentrations of 1.76 mg/mL and 0.88 mg/mL for some bacteria, MBC exceeded the maximum concentration selected for each strain. Therefore, although a concentration of GLA is sufficient to inhibit bacterial growth, higher concentrations are necessary to manifest the bactericidal effect. The same results were observed for MSG against all selected bacterial strains. The same trend was observed for GLADE-higher MBC values versus MIC values when assessed against Gram-positive bacteria and *P. aeruginosa* (but with values in the tested concentration range); the MIC value was identical to the MBC value for *K. pneumoniae* and *E. coli*.

### 3.2. The Influence on Biofilm Formation

Bacteria can be found in both planktonic and biofilm states in the environment. Bacterial biofilms are described as fixed bacterial populations enclosed in self-produced extracellular polymers, capable of living and reproducing as a collective entity known as a colony [23,24,25,26]. These structured bacterial community cells use a variety of mechanisms to coordinate activity within the community and across species [27]. The biofilm matrix, usually consisting of exopolysaccharides, nucleic acids, proteins, and lipids, helps the bacterial cells within biofilms adhere to each other but also to a substrate or material surface [10,23,28,29]. In addition to providing structural support for biofilms, this matrix protects the cells from environmental stressor agents, like antibiotics, and host immune system attacks, offering them physical and chemical protection [10]. As biofilms adhere to biological and non-biological surfaces, biofilm-forming bacteria are 10–1.000 times more resistant to antimicrobial agents compared to planktonic cells [30]. In addition to producing extracellular polymeric substances, biofilm-forming cells express different sets of genes from those of planktonic cells [10] and have the ability to avoid phagocytosis by macrophages and neutrophils [30]. Therefore, biofilm-related infections greatly contribute to nosocomial infections, including device and indwelling catheter infections (urinary tract infections, breast implant infections, periprosthetic joint infections, contact lens infections, ventilator-associated pneumonia but also dental infections, infective endocarditis, or chronic wound infections) [31]. Furthermore, biofilm infections present high resistance to antibiotic therapies, making biofilms difficult to eradicate [26].

The effect of amino acids on biofilm formation is controversial since experimental results indicate their potential anti-biofilm role but also solid supporting arguments for their involvement in biofilm formation. Regarding the biofilm formation of *S. mutans*, *D*-GLA or *L*-GLA enantiomers can inhibit biofilm formation at 40 mM; the mixture of GLA, Asp, and cysteine (the same type of enantiomer) led to a stronger inhibitory effect than individual amino acids [6]. At a concentration series of 2.5 mM, 5 mM, 10 mM, 20 mM, and 40 mM of *D*-GLA and *D*-Asp, the anti-biofilm activities of the individual amino acids and their mixture (equimolecular amounts) were evaluated. A concentration-dependent anti-biofilm (*S. aureus*) activity was observed for amino acids used separately and in combination. By combining the two amino acids, an increased anti-biofilm activity was noticed. A similar effect was observed for the mixture of *L*-amino acids in the same concentrations. Targeting the biofilm matrix components could be one of the mechanisms [15]. Also, *D*-Tyrosine demonstrated an anti-biofilm effect in the case of *P. aeruginosa* and *Bacillus subtilis* [32].

Contrary to the previously presented studies, amino acids are involved in development and cell survival, including bacteria. Thus, a hypothesis can be formulated regarding amino acids’ contribution to the stimulation of biofilm formation.

Metabolic processes within the biofilm are different, with consequences on the gradient of assimilated nutrients and metabolites destined for elimination. Biofilm development involves complex architectures and structural associations between inner and peripheral zones, forming these metabolic gradients [33]. The complex structure of biofilms depends on various resources and significant amino acid supplements. They can have recycled proteins as a source of provenance, but an adaptive increase in the genes that encode specific amino acid transporters was also observed, especially of GLN. This phenomenon influences biofilm growth through dynamic concentration gradients across the colony. The result is the modulation of the cells’ metabolic activity for nutrient diffusion in the inner part of the biofilm [34]. Additionally, bacteria are considered to tolerate exogenous *D*-amino acids, which are involved in bacterial homeostasis [7]. Table 4 summarizes information about the possible contribution of several amino acids to the biofilm development of several bacterial strains.

Therefore, greater emphasis is placed on GLA and GLN metabolism within the biofilm formation process of several bacterial strains, resulting in the biofilm growth-stimulating effect.

In the present study, the lack of an evident influence on biofilm formation and the tendency to stimulate its formation were predominantly observed at different concentrations. There are differences in the stimulation intensity depending on the bacterial strain and the tested compound.

In general, the four compounds tend to stimulate biofilm development, and where this effect is not visible, the results indicate a lack of influence on this process. A strong stimulatory effect (high Δ-Index) was observed in the case of Gram-negative bacteria, especially for *K. pneumoniae* and *P. aeruginosa.* The particular morphological characteristics of Gram-negative bacteria compared to Gram-positive ones could explain this strong stimulatory effect. The lipophilic outer membrane in Gram-negative bacteria restricts the access of hydrophilic drugs through porins (protein channels for substance passage) to a level where the drug concentration no longer has a bactericidal effect [41]. On the other hand, MSG demonstrated a weak inhibitory effect against the MRSA strain (a Δ-Index of 0.74 at 25% of the maximum tested concentration (28 mg/mL)).

GLN has been shown to have a predominantly stimulatory effect on Gram-positive and Gram-negative bacteria. The stimulatory capacity of GLN appears to be higher at lower concentrations when tested on MSSA and at the maximum concentration for MRSA. GLA showed its general stimulatory effect on biofilm formation, with the highest Δ-Index values for *P. aeruginosa* and in the case of *K. pneumoniae.* MSG did not significantly influence the biofilm development of Gram-positive bacteria, except for concentrations of 12.5% (14 mg/mL) and 6.25% (7 mg/mL) of the maximum for MRSA and *E. faecalis* (stimulatory effect). A stimulatory, outstanding impact was noticed for *P. aeruginosa*, with a Δ-Index of 11.88 at 25% of the maximum concentration (28 mg/mL). The stimulatory effect of the biofilm formation in Gram-negative bacteria was less pronounced for GLADE, being absent in the case of *E. coli*.

### 3.3. Key Aspects of Glutamic Acid (GLA) and the Glutamine (GLN) Effect on Bacterial Growth and Biofilm Formation

According to the results, GLN shows a poor inhibitory effect on bacterial growth for the chosen concentration range for MIC determination, whilst GLADE provides promising results for further studies, such as the structural optimization of the molecule. This derivative has also been highlighted for its good results in MBC determination for the chosen concentration range. Regarding the influence on biofilm formation, a generally and predominantly stimulatory effect on its development was observed, compared to the inhibitory one.

An interesting point is why GLA and MSG might produce different biological effects despite both existing mainly as glutamate at the pH (6–7) of the bacterial media used in our tests. While we agree that their ionization states are similar in these conditions, the divergence in their effects can be attributed to other physicochemical and structural factors. In the following, we issue the following hypotheses. First, MSG is a monosodium salt of glutamic acid, introducing a sodium ion into the environment. Sodium ions may influence bacterial growth and biofilm dynamics differently compared to the free acid form of GLA. The ionic strength and osmotic balance contributed by sodium ions may play a role, especially in biofilm formation and bacterial membrane interactions. Secondly, the presence of the sodium ion in MSG alters the solubility and transport dynamics compared to GLA. Bacteria may process MSG differently due to sodium-assisted transport mechanisms, which could lead to variations in uptake and metabolic integration. Lastly, structural analogues like MSG might impact bacterial enzymes, membrane transporters, or metabolic pathways differently. These differences could account for the observed variations in their antibacterial and anti-biofilm activities despite their similar state at the tested pH range. This explanation could clarify why GLA and MSG might demonstrate distinct biological effects.

Amino acids are an intriguing biological paradox with great potential in the drug development process: on the one hand, they are necessary for microbial growth and metabolism, but on the other hand, the optimization of their composition and concentration can confer anti-biofilm and antimicrobial effects. Concerning the potential antibacterial effects of GLA derivatives, most amino acid-based antibacterial agents target enzymes involved in the biosynthesis of peptidoglycan (murein), the primary component of bacterial cell walls. *D*-alanine, *D*-GLA, and *D*-GLN are amino acid residues in peptidoglycan’s peptide structure. Therefore, the hypothesis is that the enzymes responsible for their formation and processing become promising targets for antibacterial agents. GLA-based derivatives that presented inhibitory activity on MurE (sulfonamides of GLA) and MurD (naphthalene-*N*-sulfonyl-*D*-Glu) from *E. coli* and other examples of synthesized GLA-based β-lactamase inhibitors are presented in the paper of Nowak et al. (2021) [13].

Moreover, *D*-amino acids have been shown to suppress and disperse microbial biofilms; they were reported to inhibit biofilm development in *S. aureus* and *S. mutans*. Microbial cells attach to the biofilm via cellulose fibres incorporated in microbial peptidoglycan. The inclusion of *D*-amino acids into peptidoglycan (amid its synthesis) disturbs the sequence of constituent amino acids and leads to microfibre–microbial cell association alteration, therefore scattering the biofilm and releasing the sessile microbial cells [41].

A successful method to highlight the effects of amino acids is to use them as drug adjuvants or excipients, with improvements in the drug efficacy as a consequence: the salt formation technique is a method of enhancing the solubility of drugs by using amino acids; through salt formation, they can also positively impact the membrane permeability of drugs by increasing their hydrophilicity (e.g., Asp and ciprofloxacin) and stability, facilitating the drug permeation across the cellular barriers [41]. Experimental studies have demonstrated that a synergistic effect between *D*-GLA or *L*-GLA, as well as *L*-GLN, and antibiotics such as gentamicin, amoxicillin, rifampicin, quinolones, aminoglycosides, or tetracyclines can enhance antibacterial or anti-biofilm activity. Compared to the individual administration of the antibiotics, this enhanced effect is observed, likely due to various underlying mechanisms [6,7,8,9,15,42].

Conversely, the experimental findings detailed in Table 4 regarding the potential mechanism through which amino acids may stimulate biofilm formation could represent a basis for developing a hypothesis about this effect. The metabolic activity within biofilms is spatially and temporally organized, leading to a mutual dependence between the interior and peripheral cells [43]. The development and survival of biofilms depend heavily on the complex spatial connections between the inner and outer cells. The consequence of the cells’ differential metabolic processes is the formation of nutrient concentration gradients. Thus, cells growing in different areas of the biofilm consequently present a high variability from one another. Different metabolic requirements are thought to exist inside the biofilm: the inner cell mass uses lactic acid, while the dividing cells at the edges require GLN. The peripheral areas of the biofilm rely, thus, on the tricarboxylic cycle (TCA) [27].

Therefore, the biofilm metabolism involving GLN and GLA is tightly connected to biofilm formation and survival [10]. The primary amino acid donors for all nitrogen-containing metabolites and compounds, including other amino acids and DNA components, are GLA and GLN [11]. Both play a key role in forming nitrogen-rich molecules and maintaining the osmotic balance in bacteria [37]. GLA is the general amino group donor for nitrogen-containing compound synthesis, such as nucleotides, amino acids, and polyamines [10,26]. Also, GLN has an important role in supplying nitrogen in nitrogen-limiting conditions [44], and the nitrogen availability in bacterial cells is quantified by the intracellular GLN/GLA ratio [26].

In bacteria, GLN is either biosynthesized or absorbed from the surrounding environment. Its metabolism is closely regulated by the cell’s nitrogen levels and necessity [37]. The synthesis of GLN from GLA and ammonium is mediated by GS [44]. GS activity directly correlates to the energy-deriving TCA through *α*-ketoglutarate [37]. The concentration of intracellular GLA is typically higher than that of GLN, with the excess of GLN being converted into GLA by glutamate synthase [26]. Hence, GLN is essential for bacterial biofilm development, and its metabolic pathways could be a valuable target for anti-biofilm drugs [27]; an alternative could be the GS inhibitors [30]. Additionally, the inhibitors of GLA synthesis could alter the biofilm’s structure and the metabolic connections between different areas of the biofilm [26].

Furthermore, these metabolic conversions are highly relevant as they correlate with the specific necessities of the central and peripheral parts of the biofilm and have created an interesting hypothesis on the functioning of biofilms. The production of ammonium in the biofilm’s inner part is limited by and triggers the consumption of GLA in the outer layers. The excess of GLA not used by the peripheral cells diffuses inward and is converted into ammonium. Then, ammonium stimulates growth in the periphery, reducing the GLA supply to the interior. This dynamic explains why peripheral cells do not simply overcome their dependence on extracellular ammonium, as doing so would lead to continuous peripheral growth, ultimately starving and killing the interior cells within the biofilm. The periodic arrest of peripheral cell growth due to extracellular ammonium restriction promotes the overall viability of the biofilm [43]. Thus, the outer layer cells’ growth is limited to avoid the intense consumption of GLA and starve the inner area, as the interior cells synthesize the ammonia used for GLN biosynthesis in the periphery [27]. Therefore, one strategy to overcome biofilms could be to promote the continuous growth of peripheral cells to starve the inner cells of biofilm; this would consequently expose the peripheral cells to become an easy target to attack [43].

Also, the Na/GLA symporter from the environment to the intracellular space is probably highly significant, as it regulates biofilm formation by controlling the intake of exogenous GLA in some bacterial strains. It could be considered that the intake of exogenous GLA can inhibit biofilm formation and that facilitating exogenous GLA intake by modulating this symporter could potentially become a therapeutic option to treat biofilm infections [26].

Due to their complex structure and functioning, biofilms exhibit a significantly higher tolerance to antimicrobial agents than planktonic cells. It is documented that there exists a positive correlation between biofilm formation and antibiotic resistance development. Bacteria in biofilms are considerably more resistant to antimicrobial agents than planktonic cells, with over 80% of bacterial infections being caused by the formation of bacterial biofilms [23,45]. The structural features of biofilms play an essential role in developing antibiotic resistance, as several components work together to hinder the effectiveness of drugs [24]. The increased antibiotic resistance is primarily attributed to the restricted diffusion of drugs through the biofilm matrix and the physiological changes in bacteria induced by the environmental conditions within the biofilm [46]. Extracellular polymeric substances produced by biofilms promote the resistance of pathogenic bacteria to the host’s adaptive and innate immune systems [45]; they can inhibit the activity of antibiotics, which disperse through biofilms by the diffusion–reaction inhibition phenomenon, causing the antibiotics to be chelated and to form complexes, or leading to their destruction through enzymatic degradation. The extracellular polymeric substances of the biofilm either slow the process of penetration or interact with the antimicrobial agent and reduce its effectiveness [24].

In addition to the impaired diffusion of the antimicrobial agents through biofilms, a slower growth rate of biofilm-associated bacteria could be another strategy. This aspect leads to a slower uptake of antimicrobial agents, resulting in suboptimal bactericidal intracellular antibiotic concentrations. Also, changes in the chemical microenvironment and microorganism differentiation similar to spore formation correlate to drug resistance [24]. Moreover, the physical proximity of the cells in the biofilm promotes the acquisition of resistance through genetically transmissible elements [46].

Quorum sensing, an intercellular microbial communication system, allows microorganisms to exchange information between cells. Microbial quorum sensing is activated by specific extracellular chemical signals, called autoinducers, such as peptides in Gram-positive bacteria. They comprise amino acids such as serine, proline, tyrosine, leucine, isoleucine, tryptophan, glycine, cysteine, threonine, glutamic acid, valine, and phenylalanine [41].

Concerning the high resistance of biofilms, traditional antibiotics are frequently ineffective against them, highlighting the need for alternative strategies to control or eradicate biofilm formation. Combination therapies, such as those involving traditional antimicrobials and biofilm-disrupting agents, as well as synergistic approaches targeting different stages of biofilm development, offer promising strategies (including the GLA/GLN–antibiotic synergism). Additionally, nanoparticles, nano-coatings, and biomaterial modifications can be explored to target and combat biofilms specifically [31].

Given the considerations above, we hypothesize that the absence of biofilm inhibition observed in our results—despite the potential antibacterial activity of certain compounds—may be attributed to the resistance of biofilms compared to planktonic cells. The role of amino acids in biofilm development may also contribute to this outcome. Further investigation is needed to explore the potential correlation between the antibacterial effects of the compounds and their impact on biofilm formation.

### 3.4. In Silico Evaluation of Glutamic Acid Diethyl Ester’s (GLADE’s) Properties as a Potential Drug Candidate

After evaluating the antibacterial effects of the four compounds, GLADE stood out as having promising bacteriostatic and bactericidal properties against the selected bacterial strains. Therefore, GLADE is a compound that may hold potential in this field, either in its current form or following structural optimization.

In this regard, we used computational methods to characterize GLADE in terms of physicochemical properties and to theoretically estimate several pharmacokinetic properties and aspects related to drug-likeness and lead-likeness, possible antibacterial activity, and toxic and adverse effects; they are presented in Table 3. This characterization was included in this study to observe the various features of GLADE that could contribute to its usage as a potential antibacterial treatment or to choosing the alternatives to optimize the compound.

The estimation of various physicochemical and pharmacokinetic properties confirmed the hydrophilicity of GLADE. Properties such as TPSA, water solubility, Log P, BBB penetrability, and skin penetration capacity were considered to support this conclusion.

TPSA serves as an indicator of liposolubility and the ability to penetrate biological membranes. It is a parameter whose values rise with the number of polar groups in a structure [47]. The value of 78.62 Å^2^ for GLADE fits between 60 and 140 Å^2^, a range considered ideal for good cellular absorption (values below 140 Å^2^ are correlated with good intestinal absorption). The evaluation of GLADE’s water solubility indicates that the compound is very soluble, according to ESOL and Ali, and soluble according to SILICOS-IT (three different methods for water solubility evaluation: the first two are topological methods implemented from Delaney J.S. (2004) (ESOL) [48] and Ali J. et al. (2012) (Ali) [49]; the third one is a fragmental method calculated by the FILTER-IT program, version 1.0.2 [50,51]). SwissADME also offers information regarding the Log P value of a compound according to five different methods, with the Consensus Log P being the average of all five predictions [51]. The value of 0.77 for Consensus Log P fits in the desirable range of 0–2, which is considered suitable for a drug; water-soluble drugs typically have negative values, whereas a high Log P can be a problem with insoluble drugs [52]. The hydrophilic character of GLADE was also confirmed by its inability to cross the BBB, according to the same platform, it is a critical factor when developing drugs targeting the central nervous system. Skin permeation was also predicted for GLADE, as it is associated with the lipophilicity of a compound; typically, low skin permeation is observed when a molecule’s log Kp is lower than −2.5 cm/s, with the value of −7.43 cm/s indicating that the skin is not permeable for GLADE [53,54,55].

The pharmacokinetic profile was further completed by evaluating gastrointestinal absorption and classifying GLADE as a compound with high absorbance. Also, the BD score indicates the likelihood that a compound will have a bioavailability greater than 10% in rats [56]. The BD score of 0.55 out of 1 is ideal and indicates that GLADE is absorbed efficiently by the body [57,58]. Additional pharmacokinetic aspects of GLADE indicate negative results for CYP450 isoform (CYP1A2; CYP2C19; CYP2C9; CYP2D6; CYP3A4) inhibition and for being the substrate of P-gp. Also, the possible sites involved in metabolism via CYP2C9, CYP2D6, and CYP3A4 were identified; a lower score indicates a higher probability of being a site of metabolism [59]. Furthermore, the potential CYP450 metabolic pathways were assessed, as CYP450 isoforms are among the most essential drug-metabolizing enzymes.

Drug-likeness and lead-likeness characteristics are essential when defining a chemical compound’s profile. Drug-likeness is a qualitative assessment of a molecule’s probability of becoming an oral drug based on its bioavailability and describes how closely a new compound resembles already-approved medications [60]. It aids in evaluating the potential success of a compound as a drug. We evaluated GLADE according to several drug-likeness rules, such as the one of Lipinski, Eagan, Ghose, Muegge, and Veber. GLADE did not violate any drug-likeness rule; the characteristics of each rule are presented by the SwissADME platform [51]. Lead-likeness refers to selecting chemical optimization starting points to have the best potential to yield drug-like candidates [61]. A lower molecular complexity, with fewer rings and RBs, a smaller MW, and a less hydrophobic characteristic are several features that lead compounds should present to be optimized, compared to drug-like molecules [62,63]. GLADE violates two rules: it has an MW lower than 250 and more than 7 RBs [64]; however, this does not mean that GLADE could not be further optimized to obtain a drug-like compound.

Regarding the biological activity of GLADE, we were interested in observing if it could present an antibacterial effect, according to in silico predictions. The Pass online platform offered theoretical results about several potential antibacterial effects based on its chemical structure. The platform expresses the result as the probability of the compound being active (Pa) for a specific effect. This parameter estimates the likelihood that the studied compound belongs to the active compound subclass based on its structural similarity to molecules most characteristic of the “active” subset in the PASS training set [65]. Therefore, the platform estimated several effects that could be classified as antibacterial activity (Table 3); however, the Pa values were not that high for either of the possible effects compared to the maximum value of 1 (100% probability that the compound is active based on the platform’s algorithm). The highest values correspond to antituberculosis (0.366) and antimycobacterial effects (0.344). This potential activity could be assessed in vitro in a future study.

Eventually, we also obtained several predictions on the toxicological status of GLADE. According to Cramer’s rule [66], it is classified as a compound with low toxicity (class I); also, the substance would not be expected to be a safety concern based on Kroes TTC [67]. Cramer’s rule evaluates if the hybrids are normal constituents of the body, contain functional groups associated with enhanced toxicity, contain elements other than C, H, O, N, and divalent S, if they are heterocyclic compounds or have an open chain, and other structural features. The threshold of toxicological concern (TTC) is a practical risk assessment tool founded on determining a human exposure threshold for all chemicals, below which the probability of significant risk to human health is extremely low [67]. Kroes TTC also considers some structural aspects that could represent a risk (for example, aliphatic azo and azoxy groups, aromatic diazo groups, nitro, and much more). The toxtree software also indicated negative results for GLADE’s genotoxic and non-genotoxic carcinogenicity and in vitro mutagenicity.

Considering all the properties evaluated in this preliminary analysis using computational methods and the obtained results, we conclude the following:

- Based on the theoretical predictions targeted in this sub-chapter, GLADE is a hydrophilic compound, but it presents good gastrointestinal absorption and BD (SwissADME platform);
- Several antibacterial effects were identified, although not presenting a very high probability; some of them could be further assessed by in vitro or in vivo studies (Pass online platform);
- The toxicity profile defined by several determinations (Toxtree software, version 3.1.0.1851) indicates that GLADE is theoretically safe.

Therefore, linking these concluding ideas with the assessment of drug-likeness and lead-likeness leads us to the hypothesis that GLADE exhibits a strong profile as a potential drug candidate or lead compound that could undergo structural optimization and be further tested in microbiological studies to evaluate its antibacterial effects, including against other bacterial strains. We mention that the pharmacokinetic properties, antibacterial activity, and toxicity/adverse effects assessed in silico were only evaluated initially to provide a preliminary and more comprehensive profile of the compound. We acknowledge that these predictions need to be validated through further studies in the future.

## 4. Materials and Methods

**Figure 6:**
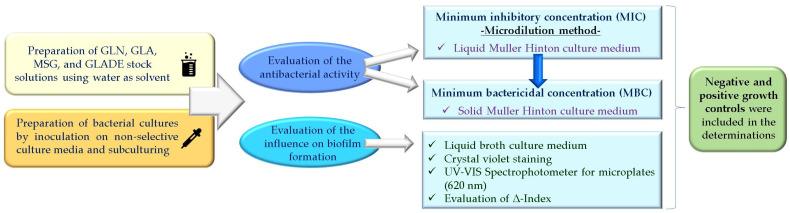
Methodological chart for the in vitro evaluation of the antibacterial activity and influence on the biofilm formation of glutamine (GLN), glutamic acid (GLA), monosodium glutamate (MSG), and glutamic acid diethyl ester (GLADE) on the selected bacterial strains.

The microbiological assessments conducted in this study for the four tested compounds (the antibacterial effect and the impact on biofilm formation) were carried out following standardized protocols for determining the minimum inhibitory concentration (MIC) and minimum bactericidal concentration (MBC), as well as for analyzing the anti-biofilm effect. The details are presented in Section 4.1 and 4.2, and Figure 6 broadly highlights the main steps followed when applying the protocols.

### 4.1. In Vitro Evaluation of the Antibacterial Activity (Minimum Inhibitory Concentration (MIC) and Minimum Bactericidal Concentration (MBC))

#### 4.1.1. In Vitro Determination of Minimum Inhibitory Concentration (MIC) by the Microdilution Method

Six bacterial strains were used for the in vitro evaluation of the MICs for the four compounds: *S. aureus* ATCC 29213 (MSSA), *S. aureus* ATCC 43300 (MRSA), *E. faecalis* ATCC 700609 (Gram-positive bacteria), *K. pneumoniae* ATCC 25922, *E. coli* ATCC 29213, and *P. aeruginosa* ATCC 27853 (Gram-negative bacteria). The Microbiology Department of the University of Medicine, Pharmacy, Science and Technology “George Emil Palade” from Targu Mures provided the strains; the strains were stocked at −70 °C before usage. Before starting the determinations, the bacteria were inoculated on non-selective culture media and subcultured.

The initial concentration (subjected to successive dilution for MIC determination) was chosen for each of the four compounds based on the scientific literature analysis as follows: *S*(+)-GLN: 2.86 mg/mL; *L*-GLA: 1.76 mg/mL; MSG: 112 mg/mL; GLADE: 102 mg/mL.

The chosen concentration ranges were determined based on several factors, including solubility constraints and data from the scientific literature on compounds like GLA, GLN, and MSG. Water was selected as the solvent for GLN and GLA to prevent interference with the experimental results, limiting the maximum stock solution concentrations to 2.86 mg/mL and 1.76 mg/mL, respectively. For MSG and GLADE, higher concentrations were possible due to their higher solubility in water (112 mg/mL and 102 mg/mL, respectively); this explains the considerable differences between their size order. These variations are linked to the fact that our purpose was to evaluate each compound separately rather than comparing their effects directly. Using purified water as a solvent did not influence the obtained results. It allowed the preparation of stock solutions (4 times more concentrated than the maximum concentration desired to be tested for each compound). Since there are no data in the scientific literature about the compound GLADE, the concentration required for the experiment was established in correlation with its high solubility in the water. The favourable results encouraged maintaining the concentration at 102 mg/mL.

All compounds were purchased from Sigma Aldrich (St. Louis, MO, USA) and Thermo Scientific (Waltham, MA, USA) (GLN ((*S*)-(+)-glutamine, CAS: 56–85-9, ID: 8.16016.0100, Batch: S6023116019, Sigma Aldrich); GLA (*L*-glutamic acid, CAS: 56–86-0, ID:1003300689, Batch: SLCK9368, Sigma Aldrich); MSG (*L*-glutamic acid monosodium salt, CAS: 142-47-2, ID: 102366233, Batch: BCCF0636, Sigma Aldrich); GLADE ((*L*-glutamic acid diethyl ester) hydrochloride, CAS: 1118-89-4, ID:459260250, Batch: A0413594, Thermo Scientific)). The producers guarantee a purity of 99%/98%. The compounds were measured using the analytical balance with a readability of up to 0.0001 g, and the solvent was measured with the automatic micropipette. Stock solutions were sterilized by filtration using 0.2 μm sterile filters.

An inoculum of 0.5 McFarland units (approximately 1.5 × 10^8^ bacterial cells/mL) was made using sterile saline solution for each targeted bacterial strain. A standard protocol for MIC determination using the liquid Mueller–Hinton bacterial culture medium was subsequently applied to the six bacterial strains and all four tested compounds.

Negative and positive controls were used for the determination. Negative controls consisted of liquid Mueller–Hinton culture medium without the bacterial inoculum and the tested compound. Positive controls were a liquid Mueller–Hinton culture medium supplemented with bacterial inoculum without the tested compound.

The microtiter plates were incubated for 24 h at 35 °C in aerobiosis. After incubation, the MIC was determined by visual examination using a mirror, according to the European Committee on Antimicrobial Susceptibility Testing (EUCAST) recommendations [68]. The experiment was performed in triplicate.

After processing the solutions for the MIC determination, we obtained a series of concentrations for each compound (corresponding to numbers 1–12 for each row of wells—Table 5).

#### 4.1.2. In Vitro Determination of the Minimum Bactericidal Concentration (MBC)

A volume of 1 μL from each well in which there was no observed bacterial growth upon the determination of the MIC was inoculated onto a solid Mueller–Hinton culture medium to determine the MBC. The plates were incubated at 35 °C for 18 h. After incubation, the MBC was determined by observing bacterial growth. Where applicable, the experiment was replicated for all four compounds on the targeted bacterial strain.

### 4.2. In Vitro Evaluation of the Influence on Biofilm Formation

The effect on biofilm formation was evaluated for all four compounds, using the same reference bacterial strains and the exact first five concentrations to determine the MIC and MBC values.

For this determination, 0.5 McFarland inoculum (approximately 1.5 × 10^8^ bacterial cells/mL) was used for each bacterium for a standard routine protocol based on crystal violet staining. Shortly, 10 μL of each inoculum was transferred into a nutrient broth (final volume of 10 mL). In sterile microtiter plates, 100 μL of bacterial suspensions were mixed with 100 μL of GLA, GLN, MSG, or GLADE (diluted in nutrient broth). The plates were incubated for 24 h at 37 °C. After incubation, the biofilms were stained using the crystal violet method (0.1% crystal violet and 30% acetic acid). The colour intensity of the wells was determined using the UV-VIS Spectrophotometer for microplates (classic EPOCH model with monochromator; 200–999 nm) at a wavelength of 620 nm (BioTek Instruments, Winooski, VT, USA). The experiment was performed in triplicate. Negative and positive growth controls were also used, prepared similarly to the ones used for MIC determination, except for the liquid broth culture medium.

The influence of the targeted compounds on the bacterial biofilm formation capacity was evaluated by comparing the average extinction obtained for each tested compound and at each concentration (following the three determinations) versus the average extinction of the positive control. The quantification was achieved by calculating a Δ-Index (the ratio between the sample mean extinction and the positive control mean extinction). This result provided information on how the tested compounds modify the biofilm formation of the bacteria included in the study. A Δ-Index value of 1 suggests no significant influence of the tested compound on biofilm formation (the biofilm formation in the sample being similar to that of the positive control). However, a variability of 25% (±0.25) is attributed to chance, so instead of the 1 value, the range is between 0.75 and 1.25. A Δ-Index greater than 1.25 suggests that the sample exhibits higher biofilm formation than the positive control, indicating an increase in biofilm formation, possibly due to the test substance. Conversely, a Δ-Index less than 0.75 suggests that biofilm formation in the sample is lower than that of the positive control, potentially implying that the test substance inhibited or reduced biofilm formation [69].

### 4.3. In Silico Evaluation of Glutamic Acid Diethyl Ester’s (GLADE’s) Properties as a Potential Drug Candidate

After assessing its antibacterial activity, GLADE emerged as a compound with promising results. Subsequently, computational methods theoretically predicted the various properties and characteristics of GLADE based on its chemical structure. The compound was characterized in terms of physicochemical properties, such as MW, HA, Csp3 fraction, RB, HAcc, HD, TPSA, water solubility (according to ESOL [48], Ali [49], and SILICOS-IT [50] methods [63]), and Log P using the platform SwissADME [51,63].

The SwissADME platform also provided predictions on several pharmacokinetic properties, such as gastrointestinal absorption, the ability to cross the BBB, to be a substrate for P-gp, and an inhibitor of some CYP450 isoforms; it also predicted a skin permeation coefficient (log Kp) calculated according to Potts R. O. et al. (1992) [70] and a bioavailability score [56,57,58]. The platform Smart Cyp version 3.0 [59] and Toxtree software version 3.1.0.1851 [71] completed the predictions of pharmacokinetic properties, indicating data on GLADE’s metabolism, such as sites involved in metabolism via CYP3A4, CYP2D6, and CYP2C9 isoforms (Smart Cyp) [72] and cytochrome P450-mediated drug metabolism (Toxtree).

The drug-likeness and lead-likeness characteristics were predicted by the platform SwissADME (number of broken rules according to Lipinsky, Muegge [73], Eagan [74], Ghose [75], and Veber [76] and the number of broken lead-likeness rules).

The possible antibacterial activity, evaluated through the probability of being active regarding the predicted antibacterial effect, was assessed using the Pass online platform version 2.0 [65].

The Toxtree software also emphasized some characteristics of toxicity based on various rules and criteria, including Cramer rules [66], Kroes TTC [67], carcinogenicity, and in vitro mutagenicity (Ames test) [77,78,79].

## 5. Conclusions

When determining the MICs for the selected six bacterial strains, GLN did not demonstrate inhibitory capacity even at the maximum tested concentration (2.86 mg/mL); inhibitory activity was observed in the case of GLA for most bacterial strains at concentrations of 1.76 mg/mL and 0.88 mg/mL; MSG inhibited the growth of all tested bacterial strains at 112 mg/mL; promising results have been obtained for GLADE, which stood out among the tested compounds with bacterial inhibitory activity on all six bacterial strains at 12.75 mg/mL and 25.5 mg/mL.

GLADE also showed satisfactory bactericidal activity for the chosen concentration range on all bacterial strains considered in the study at concentrations of 51 mg/mL and 25.5 mg/mL; this result is essential for the further optimization and evaluation of this compound.

When evaluating the influence of the tested compounds on bacterial biofilm formation, the results indicate a tendency to stimulate its development or no influence demonstrated in the process. The inhibitory effect on biofilm formation by the bacterial strains included in this study was very minimally observed, except for MSG at 25% of the maximum tested concentration (28 mg/mL) for MRSA (Δ-Index of 0.74—weakly inhibitory effect). Although the mechanism by which the compounds demonstrate their activity toward biofilm formation is unclear, the results indirectly suggest that amino acids are essential for biofilm development. This result follows the scientific literature data and supports amino acid metabolism’s importance in bacterial biofilm formation.

As GLADE stood out for its bacteriostatic and bactericidal properties, an evaluation of some of its properties using computational methods was performed. The theoretical predictions indicated that GLADE is a hydrophilic compound with good gastrointestinal absorption and BD. GLADE shows antibacterial potential, although the prediction did not show a high probability of being active. Also, it has a theoretically safe toxicity profile. Therefore, GLADE acts as a potential drug candidate or lead compound that could undergo structural optimization and be further tested in microbiological studies to evaluate its antibacterial effects, including against other bacterial strains.

The following points highlight the novelty of our research: a unique focus on GLA derivatives, the evaluation of GLA derivatives, the in silico analysis of GLADE, and mechanistic insights. While previous studies have reported the biofilm inhibition activity of amino acids, our research explicitly targets GLA and its structural analogues, including GLADE. To our knowledge, GLADE has not been extensively studied for its antibacterial and biofilm inhibition properties, making our findings particularly novel. Our study assessed GLA, GLN, MSG, and GLADE on clinically relevant Gram-positive and Gram-negative bacteria. This comprehensive approach allows us to identify a promising derivative (GLADE), for which we have conducted an in silico evaluation of physicochemical properties, pharmacokinetic profile, drug-likeness, and potential antibacterial activity. The theoretical prediction adds a new dimension to our study, suggesting that GLADE could be optimized for enhanced antibacterial effects and providing a foundation for future research.

In conclusion, GLA and some of its structural analogues are attractive options to be approached in the future for the possible antibacterial activity in a broader palette of bacterial strains, for different concentration ranges, or in combination with other antibiotics, leading to a synergistic antibacterial effect. GLADE is a promising compound, and its optimization to increase antibacterial activity could be a new research direction and an interesting approach.

## 6. Limitations

One of the difficulties of this study is related to the limited water solubility of GLN and GLA, considering that for the MIC determination, the concentration of the stock solution must be four times higher than the one subjected to successive dilutions. Purified water was the chosen solvent instead of other possible solvents (so there would be no influence on the results).

Another limitation is that the software used to estimate the targeted properties may not account for optical isomers, which could result in variations in some predicted properties.

## Figures and Tables

**Table 1:** The minimum inhibitory concentrations (MICs) of glutamine (GLN), glutamic acid (GLA), monosodium glutamate (MSG), and glutamic acid diethyl ester (GLADE) for selected bacterial strains.

Bacterial Strain	MIC (mg/mL)			
	GLN	GLA	MSG	GLADE
Gram-positive				
*Staphylococcus aureus* ATCC 29213 (MSSA)	>2.86	1.76	112	12.75
*Staphylococcus aureus* ATCC 43300 (MRSA)	>2.86	0.88	112	12.75
*Enterococcus faecalis* ATCC 700609	>2.86	1.76	112	12.75
Gram-negative				
*Klebsiella pneumoniae* ATCC 25922	>2.86	>1.76	112	25.5
*Escherichia coli* ATCC 29213	>2.86	>1.76	112	25.5
*Pseudomonas aeruginosa* ATCC 27853	>2.86	1.76	112	12.75

**Table 2:** The minimum bactericidal concentrations (MBCs) of glutamine (GLN), glutamic acid (GLA), monosodium glutamate (MSG), and glutamic acid diethyl ester (GLADE) for selected bacterial strains.

Bacterial Strain	MBC (mg/mL)		
	GLA	MSG	GLADE
Gram-positive			
*S. aureus* ATCC 29213 (MSSA)	>1.76	>112	51
*S. aureus* ATCC 43300 (MRSA)	>1.76	>112	25.5
*E. faecalis* ATCC 700609	>1.76	>112	25.5
Gram-negative			
*K. pneumoniae* ATCC 25922	-	>112	25.5
*E. coli* ATCC 29213	-	>112	25.5
*P. aeruginosa* ATCC 27853	>1.76	>112	25.5

**Table 3:** In silico theoretical prediction of physicochemical and pharmacokinetic properties, drug-likeness and lead-likeness characteristics, possible antibacterial activity, and toxic and adverse effects of glutamic acid diethyl ester (GLADE) using several platforms and software.

Evaluated Property	Predicted Results	Platform/Software
Physicochemical properties		
Molecular weight (MW)	203.24 g/mol	SwissADME
Heavy atoms (HA)	14	
Csp3 fraction (the ratio between the number of sp3-hybridized carbon atoms and the total number of carbon atoms in the molecule)	0.78	
Rotatable bonds (RBs)	8	
Hydrogen bond acceptors (HAccs)	5	
Hydrogen bond donors (HDs)	1	
Topological polar surface area (TPSA)	78.62 Å^2^	
Water solubility	Very soluble (ESOL) Very soluble (Ali) Soluble (SILICOS-IT)	
Log P	0.77 (Consensus Log P)	
Pharmacokinetic properties		
Gastrointestinal absorption	High	SwissADME
Blood–brain barrier (BBB) permeation	Negative	
Substrate for P-glycoprotein (P-gp)	Negative	
Inhibitor of CYP450 isoforms (CYP1A2; CYP2C19; CYP2C9; CYP2D6; CYP3A4)	Negative	
Skin permeation (Log Kp)	−7.43 cm/s	
Bioavailability (BD) score	0.55	
Atom reactivity (sites involved in metabolism via CYP3A4, CYP2D6, and CYP2C9 isoforms)	Cyp3A4: C8 (the carbon atom linked to the amino group) Cyp2D6: C1 (the marginal carbon atom from the ethyl group part of the ester formed with the participation of the carboxyl from the gamma position) Cyp2C9: C8	SmartCyp (version 3.0)
Cytochrome P450-mediated drug metabolism	Reactions at the primary site of metabolism: N-dealkylation Reactions at the secondary site of metabolism: amine hydroxylation Reactions at the secondary site of metabolism: liphatic hydroxylation Reactions at other sites of metabolism: O-dealkylation	Toxtree (version 3.1.0.1851); the sites of metabolism are predicted by SmartCyp and used by Toxtree
Drug-likeness and lead-likeness		
Drug-likeness	GLADE respects the rules of Lipinsky, Muegge, Eagan, Ghose, and Veber	SwissADME
Lead-likeness	2 broken rules: MW < 250 and RB > 7	
Antibacterial activity		
Antibiotic glycopeptide-like	Probability to be active (Pa) of 0.127	Pass online (version 2.0)
Antibiotic	Pa of 0.099	
Aureolysin inhibitor	Pa of 0.279	
Antimycobacterial	Pa of 0.344	
Antirickettsial	Pa of 0.291	
Anti-Helicobacter pylori	Pa of 0.267	
Bacterial leucyl aminopeptidase inhibitor	Pa of 0.196	
Aerobactin synthase inhibitor	Pa of 0.195	
Antibacterial (oftalmic)	Pa of 0.159	
Antibacterial	Pa of 0.214	
Antituberculosic	Pa of 0.366	
Antispirochetal	Pa of 0.190	
Cell wall synthesis inhibitor	Pa of 0.139	
Toxic and adverse effects		
Cramer rules	Class I (low toxicity)	Toxtree
Kroes TTC	The substance would not be expected to be a safety concern	
Carcinogenicity	Negative for genotoxic and non-genotoxic carcinogenicity	
In vitro mutagenicity (Ames test)	No alerts for *Salmonella Typhimurium* mutagenicity	

**Table 4:** The potential contribution of glutamine (GLN), glutamic acid (GLA), and poly gamma-glutamic acid (PG) to biofilm development (Ref. = references).

Amino Acid	Tested Bacteria	Observations	Ref.
GLN	*S. mutans*	GLN is a vital nitrogen source provided by its specific transporter (glutamine transport system permease protein—GlnP). The membrane transport function allows the biofilm to survive stress and benefit from favourable conditions for development.	[35]
	*S. aureus*	- The bacteria extract amino acids, including GLN, from the culture medium (MSSA and MRSA). - The enzymes involved in GLN formation, such as glutamine synthetase (GS), proved to play an essential role in *S. aureus* biofilm (the enzyme contributes to the synthesis of nitrogen derivatives and some key metabolites involved in maintaining the osmotic balance). A low level of the catalytic enzyme is associated with the suppression of biofilm formation and with the impairment of resistance against antibiotics.	[36,37]
GLA	*B. subtilis*	The biofilm-forming cells use a mechanism that directs excessive Krebs cycle metabolites to nitrogen metabolism, forming storage products. The level of some amino acids or nucleotides generated starting from GLA increases during biofilm formation.	[10]
	Gram-positive and Gram-negative bacteria	- The protective effect of GLA was evaluated on some bacterial strains subjected to the freeze-drying process, to which a concentration of 0.06 M glutamate was added at a pH of 7. Other structural derivatives of GLA were also tested, and several prevented the death of numerous Gram-positive and Gram-negative bacteria; α-Methyl-glutamic acid, N-acetyl-glutamic acid, or N-dimethyl glutamic acid are some examples. - The *D* and *L* enantiomers of GLA have shown equal intensity activities. - These effects could be due to a structural aspect—functional groups capable of forming hydrogen bonds.	[38]
GLA and GLN	*E. faecalis* and *P. aeruginosa*	Following the administration of aminooxy acetic acid (AOA) as an aminotransferase inhibitor (inhibitor of glutamate oxaloacetate transaminase and aspartate aminotransferase, resulting in glutamate and its metabolite levels’ restriction) and 6-diazo-5-oxo-*L* norleucine as a GLN analogue, high sensitivity, especially to AOA, was observed, which affected biofilm growth; the biofilm formed at subinhibitory doses of the two compounds underwent morphological changes, also being more sensitive to AOA.	[27]
PG	*B. subtilis*	PG contributes to the robustness and complexity of the biofilms’ morphology.	[39]
	*Staphylococcus* *epidermidis*	The bacterial strain produces PG that binds to antimicrobial peptides, having a protective effect against neutrophil phagocytosis.	[40]

**Table 5:** The concentrations in each well for glutamine (GLN), glutamic acid (GLA), monosodium glutamate (MSG), and glutamic acid diethyl ester (GLADE).

No. Well	Percentage of the Maximum Concentration	Concentration/Well (mg/mL)			
		GLN	GLA	MSG	GLADE
1	100%	2.860 mg/mL	1.760 mg/mL	112.000 mg/mL	102.000 mg/mL
2	50%	1.430 mg/mL	0.880 mg/mL	56.000 mg/mL	51.000 mg/mL
3	25%	0.715 mg/mL	0.440 mg/mL	28.000 mg/mL	25.500 mg/mL
4	12.5%	0.357 mg/mL	0.220 mg/mL	14.000 mg/mL	12.750 mg/mL
5	6.25%	0.178 mg/mL	0.110 mg/mL	7.000 mg/mL	6.375 mg/mL
6	3.125%	0.089 mg/mL	0.055 mg/mL	3.500 mg/mL	3.187 mg/mL
7	1.562%	0.044 mg/mL	0.027 mg/mL	1.750 mg/mL	1.593 mg/mL
8	0.781%	0.022 mg/mL	0.013 mg/mL	0.875 mg/mL	0.796 mg/mL
9	0.390%	0.011 mg/mL	0.006 mg/mL	0.437 mg/mL	0.398 mg/mL
10	0.195%	0.005 mg/mL	0.003 mg/mL	0.218 mg/mL	0.199 mg/mL
11	0.097%	0.002 mg/mL	0.001 mg/mL	0.109 mg/mL	0.099 mg/mL
12	0.048%	0.001 mg/mL	0.00075 mg/mL	0.054 mg/mL	0.049 mg/mL

## Data Availability

The original contributions presented in the study are included in the article, further inquiries can be directed to the corresponding author.

## Supplementary Materials

The following supporting information can be downloaded at: https://www.mdpi.com/article/10.3390/antibiotics14040415/s1, Table S1: The influence of glutamine (GLN) on biofilm formation, presented as Δ-Index ± standard deviation (SD), for all six bacterial strains and different concentrations of the GLN solution; Table S2: The influence of glutamic acid (GLA) on biofilm formation, presented as Δ-Index ± standard deviation (SD), for all six bacterial strains and different concentrations of the GLA solution; Table S3: The influence of monosodium glutamate (MSG) on biofilm formation, presented as Δ-Index ± standard deviation (SD), for all six bacterial strains and different concentrations of the MSG solution; Table S4: The influence of glutamic acid diethyl ester (GLADE) on biofilm formation, presented as Δ-Index ± standard deviation (SD), for all six bacterial strains and different concentrations of the GLADE solution.

## Abbreviations

AOA	Aminooxy acetic acid
BBB	Blood–brain barrier
BD	Bioavailability
*D*-Asp	*D*-Aspartic acid
GLA	Glutamic acid
GLADE	Glutamic acid diethyl ester
GLN	Glutamine
Gln	Glutamine transport system permease protein
GS	Glutamine synthetase
HA	Heavy atoms
HAcc	Hydrogen bond acceptors
HD	Hydrogen bond donors
IC50	Half-maximal inhibitory concentration
*L*-Asp	*L*-Aspartic acid
MBC	Minimum bactericidal concentration
MIC	Minimum inhibitory concentration
MRSA	Methicillin-resistant *Staphylococcus aureus*
MSSA	Methicillin-susceptible *Staphylococcus aureus*
MSG	Monosodium glutamate
MW	Molecular weight
OD	Optical densities
PG	Polygamma-glutamic acid
P-gp	P-glycoprotein
RB	Rotatable bonds
TPSA	Topological polar surface area
TTC	Threshold of toxicological concern

## References

[1] Moldovan O.-L., Vari C.-E., Tero-Vescan A., Cotoi O.S., Cocuz I.G., Tabaran F.A., Pop R., Fülöp I., Chis R.F., Lungu I.-A. (2023). Potential Defence Mechanisms Triggered by Monosodium Glutamate Sub-Chronic Consumption in Two-Year-Old Wistar Rats. Nutrients.

[2] Moldovan O.-L., Rusu A., Tanase C., Vari C.-E. (2021). Glutamate—A Multifaceted Molecule: Endogenous Neurotransmitter, Controversial Food Additive, Design Compound for Anti-Cancer Drugs. A Critical Appraisal. Food Chem. Toxicol..

[3] Oancea O.-L., Gâz Ș.A., Marc G., Lungu I.-A., Rusu A. (2024). In Silico Evaluation of Some Computer-Designed Fluoroquinolone–Glutamic Acid Hybrids as Potential Topoisomerase II Inhibitors with Anti-Cancer Effect. Pharmaceuticals.

[4] Moldovan O.-L., Sandulea A., Lungu I.-A., Gâz Ș.A., Rusu A. (2023). Identification of Some Glutamic Acid Derivatives with Biological Potential by Computational Methods. Molecules.

[5] Bhuvaneswari S., Nannu Shankar S., Nyayiru Kannaian U.P. (2015). Ajinomoto: Antibacterial Impact. Indian J. Appl. Microbiol..

[6] Tong Z., Zhang L., Ling J., Jian Y., Huang L., Deng D. (2014). An In Vitro Study on the Effect of Free Amino Acids Alone or in Combination with Nisin on Biofilms as Well as on Planktonic Bacteria of Streptococcus Mutans. PLoS ONE.

[7] Yang J., Ran Y., Liu S., Ren C., Lou Y., Ju P., Li G., Li X., Zhang D. (2024). Synergistic D-Amino Acids Based Antimicrobial Cocktails Formulated via High-Throughput Screening and Machine Learning. Adv. Sci..

[8] Fan L., Pan Z., Zhong Y., Guo J., Liao X., Pang R., Xu Q., Ye G., Su Y. (2023). L-Glutamine Sensitizes Gram-Positive-Resistant Bacteria to Gentamicin Killing. Microbiol. Spectr..

[9] Zhao X., Chen Z., Yang T., Jiang M., Wang J., Cheng Z., Yang M., Zhu J., Zhang T., Li H. (2021). Glutamine Promotes Antibiotic Uptake to Kill Multidrug-Resistant Uropathogenic Bacteria. Sci. Transl. Med..

[10] Kimura T., Kobayashi K. (2020). Role of Glutamate Synthase in Biofilm Formation by Bacillus Subtilis. J. Bacteriol..

[11] Mazloomi E., Jazani N.H., Sohrabpour M., Ilkhanizadeh B., Shahabi S. (2011). Synergistic Effects of Glutamine and Ciprofloxacin in Reduction of Pseudomonas Aeruginosa-Induced Septic Shock Severity. Int. Immunopharmacol..

[12] Tomašić T., Šink R., Zidar N., Fic A., Contreras-Martel C., Dessen A., Patin D., Blanot D., Müller-Premru M., Gobec S. (2012). Dual Inhibitor of MurD and MurE Ligases from *Escherichia coli* and *Staphylococcus aureus*. ACS Med. Chem. Lett..

[13] Nowak M.G., Skwarecki A.S., Milewska M.J. (2021). Amino Acid Based Antimicrobial Agents—Synthesis and Properties. ChemMedChem.

[14] Potapnev M.P., Andreyev S.V., Goncharova N.V., Viatkina O.I., Berdina E.L., Gapanovich V.N. (2023). Dual Effect of Amino Acid Compositions on Antibacterial Activity of Human Neutrophilic Granulocytes. Biochem. Mosc. Suppl. Ser. B.

[15] Warraich A.A., Mohammed A.R., Perrie Y., Hussain M., Gibson H., Rahman A. (2020). Evaluation of Anti-Biofilm Activity of Acidic Amino Acids and Synergy with Ciprofloxacin on Staphylococcus Aureus Biofilms. Sci. Rep..

[16] Ajayeoba T.A., Dula S., Ijabadeniyi O.A. (2019). Properties of Poly-γ-Glutamic Acid Producing-Bacillus Species Isolated From Ogi Liquor and Lemon-Ogi Liquor. Front. Microbiol..

[17] Yu Z., Wei Y., Fu C., Sablani S.S., Huang Z., Han C., Li D., Sun Z., Qin H. (2023). Antimicrobial Activity of Gamma-Poly (Glutamic Acid), a Preservative Coating for Cherries. Colloids Surf. B Biointerfaces.

[18] Ijadi Bajestani M., Mousavi S.M., Mousavi S.B., Jafari A., Shojaosadati S.A. (2018). Purification of Extra Cellular Poly-γ-Glutamic Acid as an Antibacterial Agent Using Anion Exchange Chromatography. Int. J. Biol. Macromol..

[19] Laverty G., Gorman S.P., Gilmore B.F. (2011). The Potential of Antimicrobial Peptides as Biocides. Int. J. Mol. Sci..

[20] Huan Y., Kong Q., Mou H., Yi H. (2020). Antimicrobial Peptides: Classification, Design, Application and Research Progress in Multiple Fields. Front. Microbiol..

[21] Lalonde R., Joyal C.C. (1991). Effects of Ketamine and 1-Glutamic Acid Diethyl Ester on Concept Learning in Rats. Pharmacol. Biochem. Behav..

[22] Freed W.J., Wyatt R.J. (1981). Impairment of Instrumental Learning in Rats by Glutamic Acid Diethyl Ester. Pharmacol. Biochem. Behav..

[23] Zhao A., Sun J., Liu Y. (2023). Understanding Bacterial Biofilms: From Definition to Treatment Strategies. Front. Cell. Infect. Microbiol..

[24] Sharma S., Mohler J., Mahajan S.D., Schwartz S.A., Bruggemann L., Aalinkeel R. (2023). Microbial Biofilm: A Review on Formation, Infection, Antibiotic Resistance, Control Measures, and Innovative Treatment. Microorganisms.

[25] Aliashkevich A., Alvarez L., Cava F. (2018). New Insights Into the Mechanisms and Biological Roles of D-Amino Acids in Complex Eco-Systems. Front. Microbiol..

[26] Shibamura-Fujiogi M., Wang X., Maisat W., Koutsogiannaki S., Li Y., Chen Y., Lee J.C., Yuki K. (2022). GltS Regulates Biofilm Formation in Methicillin-Resistant Staphylococcus Aureus. Commun. Biol..

[27] Hassanov T., Karunker I., Steinberg N., Erez A., Kolodkin-Gal I. (2018). Novel Antibiofilm Chemotherapies Target Nitrogen from Glutamate and Glutamine. Sci. Rep..

[28] Bessembayeva L., Kirkimbayeva Z., Biyashev B., Zholdasbekova A., Kuzembekova G., Sarybayeva D., Zhylkaidar A., Oryntaev K., Bakiyeva F. (2024). Investigation of the Antibiotic Resistance and Biofilm-Forming Ability of Staphylococcus Species from Bovine Mastitis Cases in the Almaty Region, Kazakhstan. Int. J. Vet. Sci..

[29] Eman S.I., Abdalhamed A.M., Arafa A.A., Eid R.H., Khalil H.M., Hedia R.H., Dorgham S.M., Hozyen H.F. (2024). In Vitro and in Vivo Antibacterial and Antibiofilm Efficacy of Selenium Nanoparticles against Staphylococcus Aureus Supported with Toxicopathological and Behavioral Studies in Rats. Int. J. Vet. Sci..

[30] Cui W.-Q., Qu Q.-W., Wang J.-P., Bai J.-W., Bello-Onaghise G., Li Y.-A., Zhou Y.-H., Chen X.-R., Liu X., Zheng S.-D. (2019). Discovery of Potential Anti-Infective Therapy Targeting Glutamine Synthetase in Staphylococcus Xylosus. Front. Chem..

[31] Ali A., Zahra A., Kamthan M., Husain F.M., Albalawi T., Zubair M., Alatawy R., Abid M., Noorani M.S. (2023). Microbial Biofilms: Applications, Clinical Consequences, and Alternative Therapies. Microorganisms.

[32] Su X., Cheng X., Wang Y., Luo J. (2021). Effect of Different D-Amino Acids on Biofilm Formation of Mixed Microorganisms. Water Sci. Technol..

[33] Erez A., Kolodkin-Gal I. (2017). From Prokaryotes to Cancer: Glutamine Flux in Multicellular Units. Trends Endocrinol. Metab..

[34] Caro-Astorga J., Frenzel E., Perkins J.R., Álvarez-Mena A., de Vicente A., Ranea J.A.G., Kuipers O.P., Romero D. (2020). Biofilm Formation Displays Intrinsic Offensive and Defensive Features of Bacillus Cereus. NPJ Biofilms Microbiomes.

[35] Morikawa Y., Morimoto S., Yoshida E., Naka S., Inaba H., Matsumoto-Nakano M. (2020). Identification and Functional Analysis of Glutamine Transporter in Streptococcus Mutans. J. Oral Microbiol..

[36] Zhu Y., Weiss E.C., Otto M., Fey P.D., Smeltzer M.S., Somerville G.A. (2007). Staphylococcus Aureus Biofilm Metabolism and the Influence of Arginine on Polysaccharide Intercellular Adhesin Synthesis, Biofilm Formation, and Pathogenesis. Infect. Immun..

[37] Vudhya Gowrisankar Y., Manne Mudhu S., Pasupuleti S.K., Suthi S., Chaudhury A., Sarma P.V.G.K. (2021). Staphylococcus Aureus Grown in Anaerobic Conditions Exhibits Elevated Glutamine Biosynthesis and Biofilm Units. Can. J. Microbiol..

[38] Morichi T., Irie R., Yano N., Kembo H. (1963). Protective Effect of Glutamic Acid and Related Compounds on Bacterial Cells Subjected to Freeze-Drying. J. Gen. Appl. Microbiol..

[39] Yu Y., Yan F., Chen Y., Jin C., Guo J.-H., Chai Y. (2016). Poly-γ-Glutamic Acids Contribute to Biofilm Formation and Plant Root Colonization in Selected Environmental Isolates of Bacillus Subtilis. Front. Microbiol..

[40] Kocianova S., Vuong C., Yao Y., Voyich J.M., Fischer E.R., DeLeo F.R., Otto M. (2005). Key Role of Poly-γ-Dl-Glutamic Acid in Immune Evasion and Virulence of Staphylococcus Epidermidis. J. Clin. Investig..

[41] Idrees M., Mohammad A.R., Karodia N., Rahman A. (2020). Multimodal Role of Amino Acids in Microbial Control and Drug Development. Antibiotics.

[42] Huang X., Duan X., Li J., Niu J., Yuan S., Wang X., Lambert N., Li X., Xu J., Gong Z. (2018). The Synergistic Effect of Exogenous Glutamine and Rifampicin Against Mycobacterium Persisters. Front. Microbiol..

[43] Liu J., Prindle A., Humphries J., Gabalda-Sagarra M., Asally M., Lee D.D., Ly S., Garcia-Ojalvo J., Süel G.M. (2015). Metabolic Codependence Gives Rise to Collective Oscillations within Biofilms. Nature.

[44] Ondrey J.M. (2015). The Role of Central Metabolism and Electron Transport in Biofilm Formation by Vibrio Fischeri. Master’s Thesis.

[45] Damyanova T., Dimitrova P.D., Borisova D., Topouzova-Hristova T., Haladjova E., Paunova-Krasteva T. (2024). An Overview of Biofilm-Associated Infections and the Role of Phytochemicals and Nanomaterials in Their Control and Prevention. Pharmaceutics.

[46] Folliero V., Franci G., Dell’Annunziata F., Giugliano R., Foglia F., Sperlongano R., De Filippis A., Finamore E., Galdiero M. (2021). Evaluation of Antibiotic Resistance and Biofilm Production among Clinical Strain Isolated from Medical Devices. Int. J. Microbiol..

[47] Prasanna S., Doerksen R.J. (2009). Topological Polar Surface Area: A Useful Descriptor in 2D-QSAR. Curr. Med. Chem..

[48] Delaney J.S. (2004). ESOL: Estimating Aqueous Solubility Directly from Molecular Structure. J. Chem. Inf. Comput. Sci..

[49] Ali J., Camilleri P., Brown M.B., Hutt A.J., Kirton S.B. (2012). Revisiting the General Solubility Equation: In Silico Prediction of Aqueous Solubility Incorporating the Effect of Topographical Polar Surface Area. J. Chem. Inf. Model..

[50] Winter H.D. Your Partner in Computational Drug Design. https://www.silicos-it.be/.

[51] SwissADME. http://www.swissadme.ch/.

[52] Islam M.S., Mitra S. (2023). Effect of Nano Graphene Oxide (NGO). Incorporation on the Lipophilicity of Hydrophobic Drugs. Hybrid Adv..

[53] Chauhan H.H., Chavan M.D., Choudhary R.R., Madkaikar H.M., Dalvi T.S., Shah N.J. (2022). Screening of Phytochemicals from Couroupita Guianensis as Drug Candidates against Lethal Diseases Using Insilico Analysis. Int. J. Appl. Chem. Biol. Sci..

[54] Rameshbabu S., Alehaideb Z., Alghamdi S.S., Suliman R.S., Almourfi F., Yacoob S.A.M., Venkataraman A., Messaoudi S., Matou-Nasri S. (2024). Identification of *Anastatica hierochuntica* L. Methanolic Leaves Extract-Derived Metabolites Exhibiting Xanthine Oxidase Inhibitory Activities: In Vitro and in Silico Approaches. Metabolites.

[55] Elbouzidi A., Taibi M., Laaraj S., Loukili E.H., Haddou M., Hachlafi N.E., Mrabti H.N., Baraich A., Bellaouchi R., Asehraou A. (2024). Chemical Profiling of Volatile Compounds of the Essential Oil of Grey-Leaved Rockrose (*Cistus albidus* L.) and Its Antioxidant, Anti-Inflammatory, Antibacterial, Antifungal, and Anticancer Activity in Vitro and in Silico. Front. Chem..

[56] Martin Y.C. (2005). A Bioavailability Score. J. Med. Chem..

[57] Price G., Patel D.A. (2024). Drug Bioavailability. StatPearls.

[58] Rajan R., Karthikeyan S., Desikan R. (2024). Synthesis, Structural Elucidation, In Silico and In Vitro Studies of New Class of Methylenedioxyphenyl-Based Amide Derivatives as Potential Myeloperoxidase Inhibitors for Cardiovascular Protection. ACS Omega.

[59] SMARTCyp (Version 3.0). https://smartcyp.sund.ku.dk/mol_to_som.

[60] Athar M., Sona A.N., Bekono B.D., Ntie-Kang F. (2019). Fundamental Physical and Chemical Concepts behind “Drug-Likeness” and “Natural Product-Likeness”. Phys. Sci. Rev..

[61] Polinsky A. (2008). Lead-Likeness and Drug-Likeness. The Practice of Medicinal Chemistry.

[62] Hann M.M., Oprea T.I. (2004). Pursuing the Leadlikeness Concept in Pharmaceutical Research. Curr. Opin. Chem. Biol..

[63] Daina A., Michielin O., Zoete V. (2017). SwissADME: A Free Web Tool to Evaluate Pharmacokinetics, Drug-Likeness and Medicinal Chemistry Friendliness of Small Molecules. Sci. Rep..

[64] Teague S.J., Davis A.M., Leeson P.D., Oprea T. (1999). The Design of Leadlike Combinatorial Libraries. Angew. Chem. Int. Ed..

[65] Way2Drug—Main (Version 2.0). http://www.way2drug.com/PASSOnline/index.php.

[66] Cramer G.M., Ford R.A., Hall R.L. (1976). Estimation of Toxic Hazard—A Decision Tree Approach. Food Cosmet. Toxicol..

[67] Kroes R., Renwick A.G., Cheeseman M., Kleiner J., Mangelsdorf I., Piersma A., Schilter B., Schlatter J., Van Schothorst F., Vos J.G. (2004). Structure-Based Thresholds of Toxicological Concern (TTC): Guidance for Application to Substances Present at Low Levels in the Diet. Food Chem. Toxicol..

[68] Eucast: MIC Determination. https://www.eucast.org/ast_of_bacteria/mic_determination.

[69] Ciurea C.N., Mare A.D., Mareș M., Toma F., Kosovski I.-B., Cighir A., Man A. (2024). The Influence of Farnesol and Tyrosol on Candida Spp. Virulence Traits. Germs.

[70] Potts R.O., Guy R.H. (1992). Predicting Skin Permeability. Pharm. Res..

[71] Jeliazkova N., Martinov M., Tcheremenskaia O.J.K., Networks M., Rydberg P., Avramova S., Kochev N., Jeliazkov V., Iliev L. Toxtree—Toxtree—Toxic Hazard Estimation by Decision Tree Approach (Version 3.1.0.1851). https://toxtree.sourceforge.net/.

[72] Olsen L., Montefiori M., Tran K.P., Jørgensen F.S. (2019). SMARTCyp 3.0: Enhanced Cytochrome P450 Site-of-Metabolism Prediction Server. Bioinformatics.

[73] Muegge I., Heald S.L., Brittelli D. (2001). Simple Selection Criteria for Drug-like Chemical Matter. J. Med. Chem..

[74] Egan W.J., Merz K.M., Baldwin J.J. (2000). Prediction of Drug Absorption Using Multivariate Statistics. J. Med. Chem..

[75] Ghose A.K., Viswanadhan V.N., John J. (1999). Wendoloski A Knowledge-Based Approach in Designing Combinatorial or Medicinal Chemistry Libraries for Drug Discovery. 1. A Qualitative and Quantitative Characterization of Known Drug Databases. J. Comb. Chem..

[76] Veber D.F., Johnson S.R., Cheng H.-Y., Smith B.R., Ward K.W., Kopple K.D. (2002). Molecular Properties That Influence the Oral Bioavailability of Drug Candidates. J. Med. Chem..

[77] Benigni R., Bossa C. (2011). Mechanisms of Chemical Carcinogenicity and Mutagenicity: A Review with Implications for Predictive Toxicology. Chem. Rev..

[78] Benigni R., Bossa C., Netzeva T., Rodomonte A., Tsakovska I. (2007). Mechanistic QSAR of Aromatic Amines: New Models for Discriminating between Homocyclic Mutagens and Nonmutagens, and Validation of Models for Carcinogens. Environ. Mol. Mutagen..

[79] Benigni R., Bossa C., Tcheremenskaia O. (2013). Nongenotoxic Carcinogenicity of Chemicals: Mechanisms of Action and Early Recognition through a New Set of Structural Alerts. Chem. Rev..

