# Supplementary material for: In Vitro Evaluation of the Antibacterial Effect and Influence on the Bacterial Biofilm Formation of Glutamic Acid and Some Structural Analogues
Source: Antibiotics (Basel). 2025 Apr 19;14(4):415. doi: 10.3390/antibiotics14040415 (PMC12024026; doi:10.3390/antibiotics14040415)
Supplement: Supplementary file 1 [file antibiotics-14-00415-s001.zip › antibiotics-3524121-supplementary.pdf]

antibiotics

MDPI

Article

In Vitro Evaluation of the Antibacterial Effect and Influence on the Bacterial Biofilm Formation of Glutamic Acid and Some Structural Analogues

Octavia-Laura Oancea [1], Cristina Nicoleta Ciurea [2,*], Anca Delia Mare [2], Adrian Man [2], Ruxandra Stefanescu [3] and Aura Rusu [4]

[1] Organic Chemistry Department, Faculty of Pharmacy, George Emil Palade University of Medicine, Pharmacy, Science, and Technology of Targu Mures, 540142 Targu Mures, Romania; octavia.moldovan@umfst.ro
[2] Microbiology Department, George Emil Palade University of Medicine, Pharmacy, Science, and Technology of Targu Mures, 540142 Targu Mures, Romania; anca.mare@umfst.ro (A.D.M.); adrian.man@umfst.ro (A.M.)
[3] Pharmacognosy and Phytotherapy Department, Faculty of Pharmacy, George Emil Palade University of Medicine, Pharmacy, Science, and Technology of Targu Mures, 540142 Targu Mures, Romania; ruxandra.stefanescu@umfst.ro
[4] Pharmaceutical and Therapeutic Chemistry Department, Faculty of Pharmacy, George Emil Palade University of Medicine, Pharmacy, Science, and Technology of Targu Mures, 540142 Targu Mures, Romania; aura.rusu@umfst.ro
* Correspondence: cristina.ciurea@umfst.ro

Supplementary material

Table S1. The influence of glutamine (GLN) on biofilm formation presented as Δ-Index ± standard deviation (SD) for all six bacterial strains and different concentrations of the GLN solution.

Bacteria	Δ-Index ± SD at 100% of the maximum concentration (2.860 mg/mL)	Δ-Index ± SD at 50% of the maximum concentration (1.430 mg/mL)	Δ-Index ± SD at 25% of the maximum concentration (0.715 mg/mL)	Δ-Index ± SD at 12.5% of the maximum concentration (0.357 mg/mL)	Δ-Index ± SD at 6.25% of the maximum concentration (0.178 mg/mL)
MSSA	1.44 ± 0.12 ⬆	1.36 ± 0.15 ⬆	1.42 ± 0.24 ⬆	1.62 ± 0.12 ⬆	1.61 ± 0.16 ⬆
MRSA	3.35 ± 0.17 ⬆	1.33 ± 0.16 ⬆	1.10 ± 0.18	1.78 ± 0.43 ⬆	1.92 ± 0.47 ⬆
E. faecalis	1.93 ± 0.24 ⬆	1.75 ± 0.41 ⬆	2.30 ± 0.68 ⬆	1.74 ± 0.10 ⬆	1.91 ± 0.16 ⬆
E. coli	0.85 ± 0.06	1.28 ± 0.45 ⬆	1.10 ± 0.17	1.42 ± 0.50 ⬆	1.36 ± 0.09 ⬆
K. pneumoniae	6.10 ± 0.58 ⬆	2.42 ± 0.48 ⬆	2.40 ± 0.50 ⬆	2.64 ± 0.35 ⬆	2.51 ± 0.51 ⬆
P. aeruginosa	3.77 ± 0.63 ⬆	5.09 ± 1.20 ⬆	7.89 ± 1.64 ⬆	5.79 ± 0.57 ⬆	6.75 ± 1.89 ⬆

The symbol ⬆ indicates a stimulating effect on the biofilm formation, corresponding to a Δ-Index ≥ 1.25. Unmarked values with an arrow suggest that the investigated compound does not influence the biofilm formation.

Table S2. The influence of glutamic acid (GLA) on biofilm formation presented as Δ-Index ± standard deviation (SD) for all six bacterial strains and different concentrations of the GLA solution.

Bacteria	Δ-Index ± SD at 100% of the maximum concentration (1.760 mg/mL)	Δ-Index ± SD at 50% of the maximum concentration (0.880 mg/mL)	Δ-Index ± SD at 25% of the maximum concentration (0.440 mg/mL)	Δ-Index ± SD at 12.5% of the maximum concentration (0.220 mg/mL)	Δ-Index ± SD at 6.25% of the maximum concentration (0.110 mg/mL)
MSSA	1.31 ± 0.17 ⬆	1.31 ± 0.17 ⬆	1.27 ± 0.16 ⬆	1.33 ± 0.08 ⬆	1.22 ± 0.09
MRSA	1.12 ± 0.06	1.32 ± 0.08 ⬆	0.97 ± 0.11	1.44 ± 0.07 ⬆	1.67 ± 0.16 ⬆
E. faecalis	1.05 ± 0.06	1.29 ± 0.26 ⬆	1.55 ± 0.20 ⬆	1.62 ± 0.25 ⬆	1.30 ± 0.16 ⬆

Bacteria	Δ-Index ± SD at 100% of the maximum concentration (1.760 mg/mL)	Δ-Index ± SD at 50% of the maximum concentration (0.880 mg/mL)	Δ-Index ± SD at 25% of the maximum concentration (0.440 mg/mL)	Δ-Index ± SD at 12.5% of the maximum concentration (0.220 mg/mL)	Δ-Index ± SD at 6.25% of the maximum concentration (0.110 mg/mL)
E. coli	0.83 ± 0.06	1.04 ± 0.15	1.11 ± 0.14	1.11 ± 0.10	1.26 ± 0.12 ⬆
K. pneumoniae	2.96 ± 0.87 ⬆	1.95 ± 0.20 ⬆	2.15 ± 0.23 ⬆	2.16 ± 0.22 ⬆	2.01 ± 0.29 ⬆
P. aeruginosa	1.19 ± 0.42	4.36 ± 0.50 ⬆	5.35 ± 0.56 ⬆	9.02 ± 2.79 ⬆	6.44 ± 0.82 ⬆

The symbol ⬆ indicates a stimulating effect on the biofilm formation, corresponding to a Δ-Index ≥ 1.25. Unmarked values with an arrow suggest that the investigated compound does not influence the biofilm formation.

Table S3. The influence of monosodium glutamate (MSG) on biofilm formation presented as Δ-Index ± standard deviation (SD) for all six bacterial strains and different concentrations of the MSG solution.

Bacteria	Δ-Index ± SD at 100% of the maximum concentration (112 mg/mL)	Δ-Index ± SD at 50% of the maximum concentration (56 mg/mL)	Δ-Index ± SD at 25% of the maximum concentration (28 mg/mL)	Δ-Index ± SD at 12.5% of the maximum concentration (14 mg/mL)	Δ-Index ± SD at 6.25% of the maximum concentration (7 mg/mL)
MSSA	0.94 ± 0.11	1.11 ± 0.11	1.05 ± 0.04	1.12 ± 0.09	1.19 ± 0.13
MRSA	1.01 ± 0.05	0.98 ± 0.09	0.74 ± 0.04 ⬇	1.51 ± 0.42 ⬆	1.31 ± 0.31 ⬆
E. faecalis	1.24 ± 0.11	1.21 ± 0.07	1.22 ± 0.11	1.30 ± 0.10 ⬆	1.51 ± 0.18 ⬆
E. coli	0.94 ± 0.28	0.97 ± 0.08	1.05 ± 0.19	1.21 ± 0.07	1.21 ± 0.05
K. pneumoniae	1.57 ± 0.13 ⬆	1.94 ± 0.21 ⬆	2.15 ± 0.20 ⬆	2.33 ± 0.33 ⬆	2.38 ± 0.46 ⬆
P. aeruginosa	1.38 ± 0.29 ⬆	5.64 ± 0.51 ⬆	11.88 ± 2.05 ⬆	6.55 ± 1.15 ⬆	9.38 ± 3.50 ⬆

The symbol ⬆ indicates a stimulating effect on the biofilm formation, corresponding to a Δ-Index ≥ 1.25. The symbol ⬇ indicates an inhibitory effect on biofilm formation (Δ-Index ≤ 0.75). Unmarked values with an arrow suggest that the investigated compound does not influence the biofilm formation.

Table S4. The influence of glutamic acid diethyl ester (GLADE) on biofilm formation, presented as Δ-Index ± standard deviation (SD) for all six bacterial strains and different concentrations of the GLADE solution.

Bacteria	Δ-Index ± SD at 100% of the maximum concentration (102 mg/mL)	Δ-Index ± SD at 50% of the maximum concentration (51 mg/mL)	Δ-Index ± SD at 25% of the maximum concentration (25.5 mg/mL)	Δ-Index ± SD at 12.5% of the maximum concentration (12.75 mg/mL)	Δ-Index ± SD at 6.25% of the maximum concentration (6.375 mg/mL)
MSSA	1.10 ± 0.05	1.35 ± 0.06 ⬆	1.22 ± 0.06	2.17 ± 0.31 ⬆	2.17 ± 0.31 ⬆
MRSA	1.49 ± 0.19 ⬆	1.37 ± 0.16 ⬆	0.84 ± 0.05	1.22 ± 0.14	1.56 ± 0.15 ⬆
E. faecalis	1.15 ± 0.10	1.19 ± 0.22	1.04 ± 0.06	0.97 ± 0.05	0.97 ± 0.05
E. coli	1.02 ± 0.10	0.86 ± 0.04	0.90 ± 0.08	0.89 ± 0.09	0.89 ± 0.09
K. pneumoniae	1.24 ± 0.16	1.44 ± 0.24 ⬆	1.18 ± 0.13	1.58 ± 0.24 ⬆	1.58 ± 0.24 ⬆
P. aeruginosa	1.27 ± 0.14 ⬆	1.16 ± 0.13	1.07 ± 0.10	1.00 ± 0.11	1.00 ± 0.11

The symbol ⬆ indicates a stimulating effect on the biofilm formation, corresponding to a Δ-Index ≥ 1.25. Unmarked values with an arrow suggest that the investigated compound does not influence the biofilm formation.
